# Dyspepsia Symptoms and *Helicobacter pylori* Infection, Nakuru, Kenya

**DOI:** 10.3201/eid0909.030374

**Published:** 2003-09

**Authors:** Haim Shmuely, Samson Obure, Douglas J. Passaro, Galia Abuksis, Jacob Yahav, Gerald Fraser, Silvio Pitlik, Yaron Niv

**Affiliations:** * Rabin Medical Center, Beilinson Campus, Petah Tikvah, Israel; † Rift Valley Hospital, Nakuru, Kenya; ‡ University of Illinois, Chicago, IL, USA

**Keywords:** *Helicobacter pylori*, dyspepsia, Kenya, Africa

## Abstract

The prevalence of *Helicobacter pylori* infection was studied in 138 patients with dyspepsia in a hospital in Nakuru, Kenya, and in 138 asymptomatic sex- and age-matched controls from the same population. Anti–*H. pylori* immunoglobulin (Ig) G was more prevalent in dyspeptic than asymptomatic persons (71% vs. 51%), particularly those <30 years old (71% vs. 38%). *H. pylori* seropositivity was associated with dyspepsia after adjusting for age, sex, and residence (urban or rural). Among adults, the association between *H. pylori* infection and dyspepsia remained after adjusting for the above factors and for educational attainment, family size, and manual occupation. *H. pylori* infection in asymptomatic residents of Nakuru, Kenya, was more prevalent in older persons, with a rate of 68%, than in those 31–40 years of age. However, young persons with dyspepsia had an unexpectedly high prevalence of *H. pylori* infection. *H. pylori* test-and-treat strategy should be considered in Kenyan patients with dyspepsia, particularly in persons <30 years of age.

Dyspepsia is a complex set of symptoms, rather than an indication of a specific disease, and defies simple categorization. Many causes of dyspepsia exist, including *Helicobacter pylori*. *H. pylori* may also produce different symptoms in different people. Moreover, what is known about variations in host susceptibility and *H. pylori* virulence has not been correlated with specific symptoms (*1*).

Many patients with upper gastrointestinal symptoms who seek health care do not have follow up treatment. In 60% of the investigated patients, results of tests to rule out peptic ulcer disease, gastro-esophageal reflux disease, and gastric cancer are normal, and the diagnosis is functional dyspepsia (*2*). The benefit of treatment to eradicate *H. pylori* in functional dyspepsia remains controversial (*3*,*4*). To manage uninvestigated dyspepsia in developed countries, some authors recommend screening patients >50 years of age without severe symptoms with a noninvasive test for *H. pylori,* and then treat those with positive results with *H. pylori*–eradicating drugs (*5*). However, in Africa, a disparity exists between the high prevalence of *H. pylori* infection (>90% in many areas) (*6*) and the occurrence of clinically important disease (“the African enigma”). This finding has led researchers to postulate that *H. pylori* does not play a major role in the etiologic findings of upper gastrointestinal system pathology apart, from gastritis (*7*,*8*). Thus, a noninvasive *H. pylori* test-and-treat strategy in a primary care setting in an economically depressed area, such as Africa, should be based on data that show an association between dyspepsia and *H. pylori* infection. The aim of our case-control study was to investigate the association between *H. pylori* infection and dyspepsia in Nakuru, Kenya.

## Materials and Methods

### Selection of Patients

Patients who arrived at the outpatient Gastroenterology Clinic of the Rift Valley Hospital in Nakuru, Kenya, with uninvestigated symptoms of dyspepsia for at least the previous 3 months were included in the study. Inclusion criteria were 1) presence of at least two of the following **symptoms;** **upper** abdominal pain or discomfort, bloating, nausea, vomiting, or early satiety; 2) persistent or recurrent symptoms occurring at least three times per week during >6 months in the year or years preceding the study; 3) absence of nocturnal or postprandial symptoms of gastroesophageal reflux; 4) no previous abdominal surgery except for uncomplicated appendectomy, cholecystectomy, or hernia repair.

For every dyspeptic patient, a sex- and age-matched control was recruited from a convenience sample of asymptomatic persons from the local community of Nakuru by public advertisement. Dyspepsia in the control group was excluded by clinical interview and a structured screening questionnaire.

### Gastrointestinal Symptom Questionnaire

All participants (patients and asymptomatic participants) were interviewed by one of the authors (S.O.), a local Kenyan physician, to assess symptoms. A bowel disease questionnaire formulated on the basis of a previously validated instrument (the Bowel Disease Questionnaire) was used, modified, and shortened to accommodate local Kenyan needs (*9*).

### Demographic and Socioeconomic Status

Participants were questioned about demographic data and current and childhood socioeconomic status. Age was coded into five categories (0–20, 21–30, 31–40, 41–50, and >50 years of age); among adults >21 years of age, occupation was classified as manual versus nonmanual (clerical, professional, homemaker); educational attainment as less than or at least eighth grade; number of siblings as less than or at least 7; residence as urban or rural; tobacco use as ever or never smoked cigarettes; alcohol use as less than or at least 1 L of beer or 0.5 L of wine (average 50 g ethanol) per week.

### Determination of *H. pylori* status

Whole blood was obtained from all participants. Anti-*H. pylori* immunoglobulin (Ig) G was determined with the Helisal Rapid Blood Test kit (Cortecs Diagnostics, UK). This test achieved 89% sensitivity and 91% specificity versus histologic examination and urease testing in Australian adults (*10*).

Kits were stored at 4°C and equilibrated to room temperature before use. The tests were performed according to the manufacturer’s instructions. All results were read by one of the authors (S.O.). Our laboratory recently evaluated the Helisal test in 20 Israeli adults (20–70 years of age, median 42 years of age), and demonstrated the test to be 100% sensitive and 90% specific (*11*).

### Statistical Analysis

Bivariate analyses were performed by using the Fisher exact test for categorical variables and the Student t test or Kruskal-Wallis two-sample test for integer and continuous variables. Multivariate analyses were performed by applying backwards-elimination logistic regression to all demographic and socioeconomic variables evaluated in the bivariate analyses; parsimonious models were developed, which included only age and those variables associated with a mutually adjusted p value of <0.10. Only participants >21 years of age were included in the models investigating the role of education, occupation, and family size on the *H. pylori*–dyspepsia relationship. All p values were two-tailed.

## Results

Seropositivity for *H. pylori* was found in 98 (71%) of 138 symptomatic patients and 70 (51%) of 138 asymptomatic participants (odds ratio [OR], 2.4; 95% confidence interval [CI], 1.4 to 4.0; p<0.001). In the asymptomatic participants, the prevalence of *H. pylori* infection increased with age, from 18% in the 0- to 10-year age group to 48% in the 11- to 20-year age group, peaking (68%) in the 31- to 40-year age group. In the dyspeptic patients, the prevalence of *H. pylori* infection was 60% to 73% in all age groups (Table 1). Among persons <21 years old, *H. pylori* infection was more prevalent in those with symptoms than those without (17 [71%] of 24 vs.12 [38%] of 31; OR, 4.1; 95% CI, 1.1 to 14.9; p=0.02). Similarly, *H. pylori* seropositivity showed a significant association with dyspepsia among persons 21–30 years of age (35 [73%] of 48 vs. 36 [48%] of 74; OR, 2.6; 95% CI 1.2 to 6.7; p=0.01), but not among persons >30 years of age (46 [70%] of 66 vs. 20 [63%] of 32; OR, 1.4; 95% CI, 0.4 to 2.8; p=0.8).

**Table 1:** Risk (prevalence odds ratios) of upper gastrointestinal symptoms associated with *Helicobacter pylori* infection by age, among 276 residents of Nakuru, Kenya

Age (y)	Cases^a^ (N=138) (%)	Controls^b^ (N=138) (%)	OR (95% CI)^c^	p value
0–20	17/24 (71)	12/32 (38)	4.1 (1.1 to 14.9)	0.02
21–30	35/48 (73)	36/74 (48)	2.6 (1.2 to 6.7)	0.01
31–40	27/38 (71)	15/22 (68)	1.2 (0.3 to 4.1)	1.0
41–50	13/18 (72)	3/6 (50)	2.6 (0.5 to 26.3)	0.4
>50	6/10 (60)	2/4 (50)	1.3 (0.1 to 20.2)	1.0
0–30	52/72 (72)	48/108 (44)	3.3 (1.6 to 6.5)	<0.001
>30	46/66 (70)	20/32 (63)	1.0 (0.4to 2.8)	0.8

^a^Persons with upper gastrointestinal symptoms. ^b^Persons without upper gastrointestinal symptoms. ^c^OR, odds ratio; CI, confidence interval.

On bivariate analysis, infection with *H. pylori*, older age, female sex, working as a manual laborer (>21 years of age), less education, and larger family size (>7 siblings) were associated with dyspepsia in adults (Table 2). *H. pylori* infection was associated with dyspepsia after adjusting for age, sex, and urban residence (OR, 2.0; CI, 1.1 to 3.3; p=0.02), and among adults, after adjusting for these factors and for education, family size, and occupation (OR, 2.4; 95% CI, 1.1 to 4.9; p=0.02) (Table 3).

**Table 2:** Risk factors for upper gastrointestinal symptoms among 276 residents of Nakuru, Kenya

Risk factor	Cases^a^ N=138 (%)	Controls^b^ N=138 (%)	OR (95% CI)^c^	p value
*Helicobacter pylori* infection	98/138 (71)	70/138 (51)	2.4 (1.5 to 3.9)	<0.001
Less education^d,e^	42/114 (37)	16/106 (15)	3.3 (1.71 to 6.27)	<0.001
>7 siblings^d^	77/114 (68)	44/105 (42)	2.9 (1.7 to 5.0)	<0.001
Manual laborer^d,f^	32/91 (35)	9/73 (12)	3.9 (1.7 to 8.6)	<0.001
Female gender	81/138 (59)	56/138 (41)	2.1 (1.3 to 3.4)	0.003
Alcohol use^d^	8/113 (7)	21/104 (20)	0.3 (0.1 to 0.7)	0.005
Ever smoked^d^	6/115 (7)	3/105 (3)	1.9 (0.5 to 7.0)	0.4
Urban residence^g^	86/138 (62)	80/138 (58)	1.2 (0.7 to 1.9)	0.5
Age, median (range)	30 y (1–62)	23 y (2–74)		0.001

^a^Persons with upper gastrointestinal symptoms. ^b^Persons without upper gastrointestinal symptoms. ^c^OR, odds ratio; CI, confidence interval. ^d^Adults >21 years of age. ^e^Up to 8th grade. ^f^Manual laborers versus persons in clerical or professional fields or housewives. ^g^City or town versus rural.

**Table 3:** Risk factors for upper gastrointestinal symptoms among 276 residents of Nakuru, Kenya

	All ages		Adults >21 y	
Risk factor	OR (95%CI)^a^	p value	OR (95%CI)	p value
*Helicobacter pylori* infection	2.2 (1.3 to 3.8)	0.003	2.2 (1.1 to 4.8)	0.03
Age (y)				
0–20	1.0^b^	—^b^	—^c^	—^c^
21–30	0.7 (0.4 to 1.4)	0.3	1.0 ^b^	—^c^
31–40	2.1 (1.0 to 4.7)	0.06	1.5 (0.7 to 3.4)	0.3
41–50	(1.2 to 11.4)	0.02	3.5 (1.1 to 11.1)	0.03
>50	(0.9 to 13.2)	0.06	2.0 (1.4 to 9.0)	0.4
Female gender	2.2 (1.3 to 3.7)	0.003	—^d^	—
>7 siblings	—^c^	—	3.2 (1.5 to 7.0)	0.003
Manual laborer	—^c^	—	3.5 (1.4 to 9.3)	0.003
Ever smoked	—^c^		19.4 (1.5 to 256.7)	0.02
Alcohol use	—^c^		0.3 (0.1 to 1.0)	0.05

^a^OR, odds ration; CI, confidence interval; —, not applicable. ^b^Reference group. ^c^Variable not included in all-ages model. ^d^Removed by backwards-elimination logistic regression.

This *H. pylori* serologic study in residents of the Nakuru District of Kenya found the expected relationship between *H. pylori* prevalence and age among asymptomatic participants. However, among persons with dyspepsia, the prevalence was consistently high for all ages, which yielded an unequivocal association between *H. pylori* infection and dyspepsia among persons <30 years of age.

Other studies of Africans with dyspepsia have yielded a mean prevalence of 65% (range 60% to 71%), which is consistent with our results (*12*,*13*). Our recruitment strategy was very similar to the strategy of a study conducted in Cape Town, South Africa (*13*). In that 1993 study, *H. pylori* prevalence among a subset of Africans of non-Caucasian descent with nonulcer dyspepsia attending a gastroenterology clinic was 71%, the same as in our study. However, since the South African study did not include healthy controls, no generalizations can be made about the association between *H. pylori* and dyspepsia in different part of the continent. Recently, healthy Nigerian adults and dyspeptic patients were found to have similar prevalence of *H. pylori* infection (80% vs. 88%), but the sample size (50 persons) may have been too small to detect the moderate effects found in our and other’s studies, particularly in the subgroup of persons <30 years of age (*14*).

The role of *H. pylori* in dyspepsia is poorly understood (*15*,*16*). Dyspeptic symptoms are common in sub-Saharan Africa (*17*); in some regions, they may account for up to 10% of hospital admissions (*18*). Because healthcare resources in Kenya are limited, physicians direct diagnostic tests for patients in whom a definitive diagnosis is important for treatment (e.g., those with peptic ulcer or gastric cancer). Since a large fraction of the dyspepsia in younger Africans is attributable to *H. pylori,* and since dyspepsia in this age group is likely to represent a benign process, a test-and-treat strategy may be appropriate in this age group. This approach involves *H. pylori* testing of uninvestigated dyspeptic patients without **severe** **symptoms** or signs suggestive of underlying malignancy (unexplained recent weight loss, dysphagia, hematemesis or melena, anemia, previous **gastric** surgery, and palpable mass). Those with positive results would undergo *H. pylori* eradication therapy before endoscopy is considered. Those **test**ing negative would undergo endoscopy only if dietary and behavioral maneuvers do not ameliorate the complaints.

Our study has several limitations. We did not investigate the underlying causes of dyspepsia, so we may have included an unknown number of participants with peptic ulcer disease or other organic pathology. Additional limitations include the use of convenience (self-selected) controls as a proxy for population controls and the use of prevalence ORs (to make the crude and adjusted risks commensurate) instead of risk ratios. These factors may have led us to overestimate the association between *H. pylori* and dyspepsia. On the other hand, the use of a test with imperfect sensitivity and specificity may have led us to underestimate this association.

In a recent, randomized, placebo-controlled trial in a developed country, eradication therapy proved successful in a subset of patients with nonulcer dyspepsia (*19*). However, these findings were not confirmed in another trial of similar design (*20*). This disparity suggests either that the relationship between *H. pylori* and nonulcer dyspepsia is weak or that dyspepsia is a heterogeneous disorder. Thus, the effectiveness of a test-and-treat strategy in the developing world may vary by the population studied or by biological and cultural differences in the definition of dyspepsia.

This study demonstrates that in Nakuru, Kenya, *H. pylori* infection is associated with dyspepsia, particularly in persons <30 years of age. Since solid evidence exists that *H. pylori* eradication prevents the development (*21*) and recurrence (*22*) of gastric carcinoma and promotes regression of B-cell lymphoma of the mucosa-associated lymphoid tissue (MALT) tissue of the stomach (*23*), the proposed test-and-treat strategy may be an efficient use of health resources in Kenya and perhaps other African countries.
